# Impact of Heat Stress on Ovarian Function and circRNA Expression in Hu Sheep

**DOI:** 10.3390/ani15142063

**Published:** 2025-07-12

**Authors:** Jianwei Zou, Lili Wei, Zhihua Mo, Yishan Liang, Jun Lu, Juhong Zou, Fan Wang, Shaoqiang Wu, Hai’en He, Wenman Li, Yanna Huang, Qinyang Jiang

**Affiliations:** 1 College of Animal Science and Technology, Guangxi University, Nanning 530004, China; 2218401009@st.gxu.edu.cn (J.Z.); 15977285951@163.com (L.W.); 19978391494@163.com (Z.M.); liangys2025@163.com (Y.L.); 2218391030@st.gxu.edu.cn (J.L.); zz898600@126.com (J.Z.); wangfan221215@126.com (F.W.); 15290390954@163.com (S.W.); eny-work@outlook.com (H.H.); liwenman010215@163.com (W.L.); 2 Guangxi Key Laboratory of Animal Breeding, Disease Control and Prevention, Guangxi University, Nanning 530004, China

**Keywords:** heat stress, Hu sheep, ovary, circRNA, RNA-seq

## Abstract

Climate change poses an increasing threat to livestock reproduction, with heat stress (HS) known to significantly impair ovarian function. This study aimed to elucidate the impact of HS on ovarian function and circRNA expression profiles in Hu sheep. Twelve ewes were randomly assigned to a control (Con, *n* = 6) or HS group (*n* = 6) and exposed to different temperatures for 68 days. Compared with the Con group, HS significantly increased the respiratory rate (108.33 ± 3.72 vs. 63.58 ± 2.42 breaths/min), pulse rate (121.17 ± 3.98 vs. 78.08 ± 3.31 beats/min), and rectal temperature (40.17 ± 0.14 °C vs. 39.02 ± 0.21 °C; *p* < 0.05). Concurrently, serum antioxidant levels were markedly decreased, including total antioxidant capacity (T-AOC), total superoxide dismutase (T-SOD), and glutathione peroxidase (GSH-Px) (*p* < 0.05). Histological analysis revealed a significant reduction in the numbers of primordial, primary, secondary, and mature follicles, alongside an increase in antral follicles (*p* < 0.05). TUNEL staining demonstrated enhanced granulosa cell apoptosis (*p* < 0.05), accompanied by the upregulation of pro-apoptotic genes *Bax* and *Caspase-3* and downregulation of the anti-apoptotic gene *Bcl-2*, as confirmed by qPCR (*p* < 0.05). CircRNA sequencing identified 152 differentially expressed circRNAs (120 upregulated, 32 downregulated), and enrichment analyses indicated their involvement in apoptosis, mitophagy, and the FoxO signaling pathway. Collectively, these findings demonstrate that HS impairs ovarian physiology and antioxidant defense, induces follicular damage and cell apoptosis, and alters circRNA expression profiles, providing new insights into the molecular mechanisms underlying HS-induced reproductive dysfunction in Hu sheep.

## Simple Summary

Heat stress is a common environmental factor that adversely affects the reproductive health of sheep. In this study, Hu sheep were used as a model to investigate the effects of heat stress on ovarian function. The results showed that heat stress impaired follicular development in the ovaries, reduced antioxidant capacity, and promoted the apoptosis of ovarian granulosa cells. Further circular RNA sequencing revealed that several key circRNAs were involved in stress responses and cell death pathways. This study contributes to the understanding of the molecular mechanisms by which heat stress damages ovarian function and provides a theoretical basis for improving heat tolerance in sheep breeding.

## 1. Introduction

With global warming, heat stress (HS) poses an increasing threat to the sheep industry worldwide [1]. Studies have shown that HS disrupts sheep physiology, alters heat shock protein gene expression, and impairs antioxidant capacity, growth, and reproduction [2,3,4,5]. In summer, sheep show increased respiratory rate, heart rate, rectal temperature, and oxidative stress levels compared to winter [6], along with significantly reduced lambing rates [7]. Heat stress reduces healthy ovarian follicles, leading to decreased fertility [8,9]. Sheep are one of the most important livestock species globally, with a population exceeding 1.2 billion worldwide [10]. More than 40% of the global sheep population is raised in Asia, and China is among the leading sheep-producing countries [11]. Hu sheep (Ovis aries), widely raised in southern China, are known for their strong reproductive performance and adaptability [12]. However, frequent high temperatures during the summer in southern regions severely threaten their fertility. Therefore, understanding the effects of heat stress on Hu sheep and their underlying mechanisms is crucial for improving their heat tolerance. Despite the significant economic and social value of Hu sheep in China, molecular studies on how heat stress affects their ovarian function remain limited.

With technological advancements, omics approaches have become key tools for exploring biological phenotypes, gene expression, and regulatory mechanisms [13]. Various omics approaches, including transcriptomics [14], metabolomics [15], and microbiomics [16,17], have been widely applied to investigate heat stress in sheep, revealing associated physiological changes and underlying molecular mechanisms. Transcriptome sequencing enables comprehensive analysis of gene expression and regulation under specific conditions [18]. Circular RNAs (circRNAs), a class of stable, non-coding RNAs with tissue-specific expression patterns, typically ranging from 200 to 1000 nt [19,20], have attracted considerable attention due to their diverse regulatory roles in biological processes [21,22,23]. Studies suggest that circRNAs play critical roles in growth [24], meat quality [25], and reproduction [26] in livestock. Thus, identifying HS-related circRNAs and pathways in Hu sheep ovaries via circRNA-seq offers novel insights into the molecular basis of HS responses.

In recent years, circRNA-seq has been widely applied in livestock research to identify HS-related circRNAs and signaling pathways in pigs [27] and cattle [28]. For example, circRNA-seq of mammary tissues from dairy cows under summer HS and winter non-stress conditions revealed that differentially expressed circRNAs (DE circRNAs) were mainly enriched in lipid metabolism-related pathways [29]. In pig pituitary tissues, 59 DE circRNAs were identified between heat-stressed and non-stressed groups, some of which may regulate pituitary-specific gene expression [30]. Moreover, circRNAs associated with thermotolerance were identified in the peripheral blood of heat-tolerant and heat-sensitive cows, including circRNA3685, which interacts with bta-miR-138 to target HIF1A and potentially modulate lactation function under HS [31]. However, studies on circRNAs related to HS in sheep remain limited. Based on previous studies in other species showing that heat stress significantly impairs reproductive performance in livestock and poultry, with circRNAs playing key regulatory roles in this process, we hypothesize that circRNAs may serve important regulatory functions in the ovarian response to heat stress in Hu sheep.

Therefore, this study aims to establish a heat stress model in Hu sheep to compare changes in physiology, antioxidant capacity, ovarian tissue damage, and gene expression between heat-stressed and control groups, with the goal of gaining a deeper understanding of the impact of heat stress on reproductive function in Hu sheep. Additionally, we aim to identify candidate circRNAs affected by heat stress and analyze their functional roles in ovarian tissue, in order to elucidate the molecular mechanisms underlying heat stress-induced ovarian damage in Hu sheep and provide new insights and molecular targets for improving heat tolerance in Hu sheep.

## 2. Materials and Methods

### 2.1. Ethics Statement

All experimental procedures involving animals were conducted in accordance with the guidelines of the Institutional Animal Care and Use Committee (IACUC) and were approved by the Animal Ethics Committee of Guangxi University (approval No. GXU-2023-0135).

### 2.2. Experimental Site and Environmental Conditions

This study was conducted from July to September 2023 at the Hu sheep breeding base of Guangxi Anxin Animal Husbandry Co., Ltd., located in Dahua County, Hechi City, Guangxi Zhuang Autonomous Region, China (24.04° N, 107.33° E; elevation ~340 m). The region has a subtropical monsoon climate, with an average summer daylight duration of 13–14 h, an average temperature of approximately 28–32 °C, a maximum temperature of 38–40 °C, and relative humidity ranging from 75% to 85%.

### 2.3. Animals and Experimental Diets

**Figure 1:**
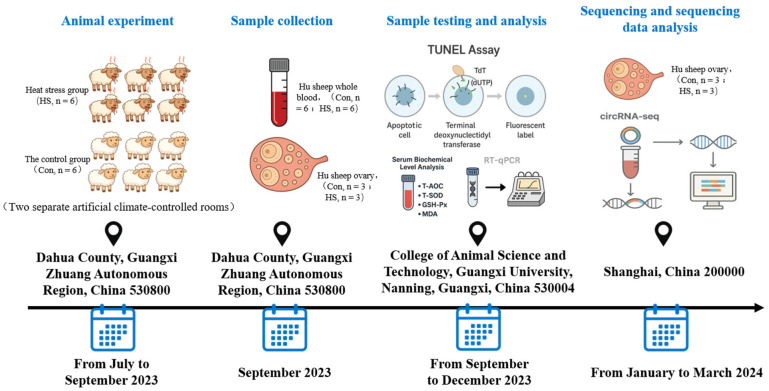
Schematic diagram of the experimental protocol.

The experimental animals used in this study were Hu sheep, a non-seasonal estrus breed of sheep that can exhibit estrus and be bred throughout the year, with regular estrous cycles and clearly observable estrus behavior. Twelve clinically healthy, 2- to 3-year-old multiparous Hu ewes with similar body weights (41.69 ± 1.17 kg) that had recently completed estrus were selected from the Hu sheep breeding base of Guangxi Anxin Animal Husbandry Co., Ltd. and randomly assigned to the control group (Con, *n* = 6) or the heat stress group (HS, *n* = 6). The animals were housed in two separate artificial climate-controlled rooms. The control group was maintained at a constant temperature of 23 °C throughout the day, while the HS group was exposed to 38 °C from 08:00 to 18:00 and 28 °C from 18:00 to 08:00 the next day. This time-spanning experimental protocol is illustrated in Figure 1. Both groups were fed the same diet and had ad libitum access to water. The composition and nutritional values of the feed are provided in Table 1. The trial lasted for 68 days. Ambient temperature and humidity were recorded every 30 min using an electronic temperature and humidity data logger RC-4 (Jingchuang Electronics, Xuzhou, Jiangsu, China). The temperature–humidity index (THI) was calculated using the following formula: THI = (1.8 × Tdb + 32) − (0.55 − 0.0055 × RH) × (1.8 × Tdb − 26.8) [32], where Tdb is the dry-bulb temperature (°C) and RH is the relative humidity (%). Respiratory rate (RR), pulse rate (PR), and rectal temperature (RT) were measured every 7 days at 14:30. Estrus detection was conducted twice daily using teaser rams, with each observation lasting more than 30 min.

### 2.4. Sample Collection

At the end of the experiment, blood samples were collected from the jugular vein of the Hu ewes (*n* = 12) 12 h after the onset of estrus. Serum was separated by centrifugation at 3000× *g* for 15 min at 4 °C and stored at −80 °C for further analysis. In addition, according to the Chinese Agricultural Standard NY/T 3469-2019, three 2- to 3-year-old multiparous Hu ewes from each group were randomly selected and slaughtered 12 h after estrus. Ovarian tissues were harvested, halved, and processed: one portion was fixed in 4% paraformaldehyde for histological analysis (*n* = 6); the other was snap-frozen in liquid nitrogen for circRNA analysis (*n* = 6).

### 2.5. Serum Biochemical Level Analysis

The concentrations of several important antioxidant capacity indices were detected in the serum samples of sheep (*n* = 12). The antioxidant capacity indices included total antioxidant capacity (T-AOC), total superoxide dismutase (T-SOD), glutathione peroxidase (GSH-Px), and malondialdehyde (MDA). According to the manufacturer’s protocol, detection kits (Nanjing Jiancheng Bioengineering Institute, Nanjing, China) were used to detect the antioxidant capacity in the serum.

### 2.6. Histological Assay

Ovarian tissues (*n* = 6) fixed in 4% paraformaldehyde were processed by Servicebio (Wuhan, China). Samples were dehydrated using a graded ethanol series (75–100%), cleared in xylene (50–100%), embedded in paraffin, and sectioned at 5 μm thickness. Sections were deparaffinized and stained with hematoxylin and eosin (H&E). Follicle development was evaluated under an Olympus DP71 microscope. Images were analyzed using CaseViewer 2.4.0 (3DHISTECH, Budapest, Hungary).

### 2.7. TUNEL Assay

Ovarian sections (5 μm) were incubated with citrate buffer for antigen retrieval (8 min), followed by TUNEL reaction mix at 37 °C in the dark for 1 h. DAPI (Servicebio, Wuhan, China) was used to counterstain nuclei for 10 min. Slides were observed under a Zeiss fluorescence microscope, with at least 9 fields captured per replicate. TUNEL-positive cells were quantified, and apoptosis rates were calculated using ImageJ 1.53t.

### 2.8. RNA Extraction, Small RNA Library Construction, and Sequencing

The ovarian tissues of 3 randomly selected ewes from each group were sent to Shanghai Personal Biotechnology Co., Ltd. (Shanghai, China) for circRNA-seq. Total RNA was treated with the Epicenter Ribo-Zero rRNA Removal Kit to remove rRNA, and then digested with RNase R. RNA was fragmented (~300 bp) and reverse-transcribed using random hexamers to generate cDNA. After second-strand synthesis, 400–500 bp fragments were selected using AMPure XP beads, PCR-amplified, and purified again. Library quality was assessed with an Agilent 2100 Bioanalyzer System (G2939BA, Agilent Technologies, Santa Clara, CA, USA). Libraries were sequenced using paired-end (PE) mode on an Illumina HiSeq platform (Personalbio, Shanghai, China).

### 2.9. Statistical Analyses

Raw data in FASTQ format were quality-checked using FastQC v0.11.9. Clean reads were evaluated for Q20, Q30, and GC content. Reference genome and annotation files were downloaded from NCBI (GCF_016772045.2_ARS-UI_Ramb_v3.0). CircRNA detection was performed using Find_circ and CIRI2. Circos plots were generated using Circos 0.69-6. Expression levels were normalized to TPM (transcripts per million), and differential expression analysis was conducted using DESeq v1.10.1. Significantly differentially expressed circRNAs were defined as those with adjusted *p*-values (*p* adj) < 0.05. Gene Ontology (GO) functional enrichment analysis and Kyoto Encyclopedia of Genes and Genomes (KEGG) pathway enrichment analysis were performed on the differentially expressed host genes of circRNAs using clusterProfiler (v4.6.0). GO terms and KEGG pathways with adjusted *p*-values (*p* adj) < 0.05 were considered significantly enriched.

### 2.10. RT-qPCR Validation

Eight circRNAs (six upregulated, two downregulated) were selected for RT-qPCR validation. Total RNA was extracted using TRIzol (Vazyme, Nanjing, China) and quantified by spectrophotometry (Miulab Instruments, Hangzhou, China). Reverse transcription was performed using PrimeScript™ RT Master Mix (TaKaRa, Otsu, Japan). Primers were designed using Primer Premier 6.0 (see Supplementary Table S1). RT-qPCR was carried out on a Bio-Rad CFX96 system. Relative expression was calculated using the 2^−ΔΔCt^ method, with GAPDH as the internal control.

### 2.11. Statistical Analysis

All data were analyzed using SPSS 26.0 software. The Shapiro–Wilk test was used to assess data normality. Normally distributed data are expressed as the mean ± standard error of the mean (SEM), and differences between groups were analyzed using independent-sample *t*-tests. For non-normally distributed data, the Mann–Whitney U test was applied. A *p*-value of <0.05 was considered statistically significant. Dependent variables included serum antioxidant indices, gene expression levels, and follicle counts; the treatment group was used as the independent variable.

## 3. Results

### 3.1. The Effects of HS on the Physiological Characteristics of Hu Sheep

**Figure 2:**
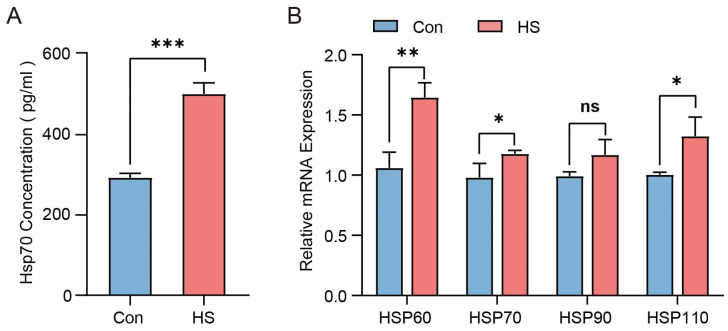
The effect of heat stress on the HSP family in Hu sheep. (**A**) Concentration of *HSP70* in the serum of Con- and HS-group sheep. (**B**) Relative expression patterns of *HSP* genes in ovarian tissues of Con and HS groups. ns, not significant; * *p* < 0.05; ** *p* < 0.01; *** *p* < 0.001.

As shown in Table 2, the average temperature–humidity index (THI) in the barn of the HS group was 91.54 ± 0.93, which was higher than that of the Con group (69.36 ± 1.9; *p* < 0.05). Meanwhile, the respiratory rate (RR), pulse rate (PR), and rectal temperature (RT) of Hu sheep in the HS group were also higher than those in the control group (*p* < 0.05). In addition, compared with the Con group, the serum *HSP70* level was significantly elevated in the HS group (*p* < 0.001), and the mRNA expression levels of *HSP60*, *HSP70*, and *HSP110* in ovarian tissues were also significantly higher in the HS group (*p* < 0.05), while *HSP90* showed no significant difference (Figure 2A,B). These results indicate that Hu sheep in the HS group experienced severe heat stress, whereas those in the Con group did not exhibit obvious signs of heat stress.

### 3.2. Effects of HS on Antioxidant Capacity in Hu Sheep

**Figure 3:**
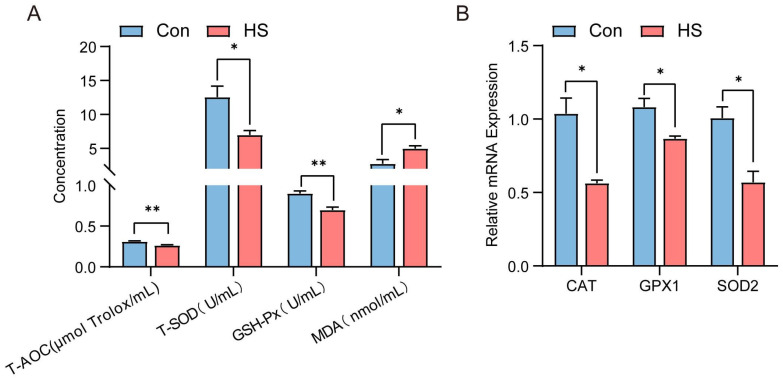
Effect of heat stress on antioxidant enzyme levels in serum and mRNA expression of antioxidant genes in ovaries of Hu sheep. (**A**) Concentrations of T-AOC, T-SOD, GSH-Px, and MDA in the serum of Con- and HS-group sheep. (**B**) Relative expression levels of mRNAs of antioxidant-related genes (*CAT*, *GPX1*, and *SOD2*) in ovarian tissues of Hu sheep in the Con group and the HS group. ns, not significant; * *p* < 0.05; ** *p* < 0.01.

Compared to the control group, the heat-stressed (HS) group of Hu sheep exhibited lower serum levels of total antioxidant capacity (T-AOC), total superoxide dismutase (T-SOD), glutathione peroxidase (GSH-Px), and malondialdehyde (MDA) (*p* < 0.05, Figure 3A). Meanwhile, the mRNA expression levels of antioxidant-related genes (*CAT*, *GPX1*, and *SOD2*) in ovarian tissues were also lower than those in the control group (*p* < 0.05, Figure 3B).

### 3.3. Effects of HS on Ovarian Tissue Morphology and Cell Apoptosis in Hu Sheep

**Figure 4:**
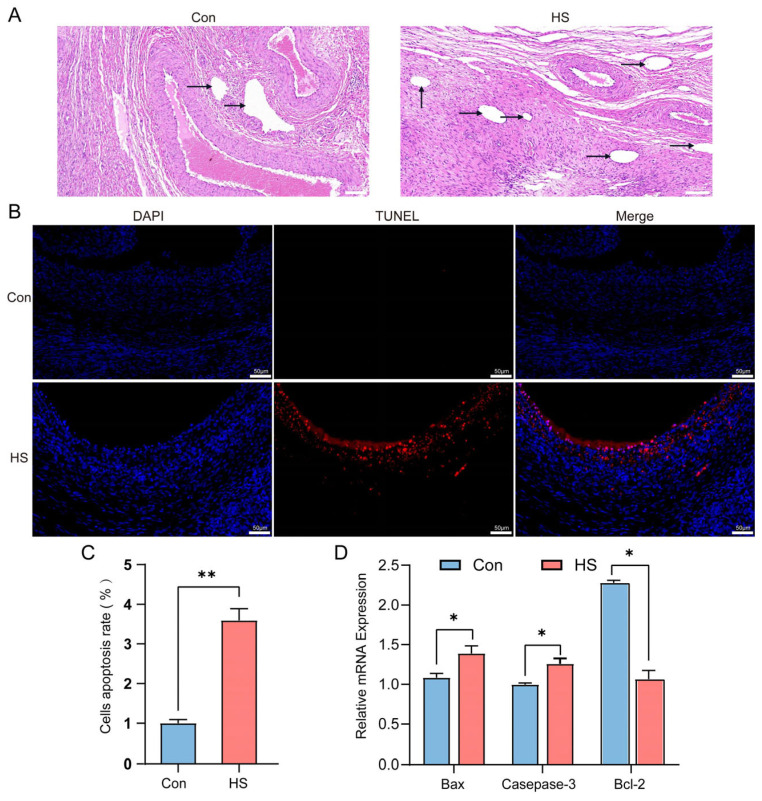
Effect of heat stress on ovarian follicle development, apoptosis, and mRNA expression of apoptosis-related genes in the ovaries of Hu sheep. (**A**) HE staining of ovarian tissues from the Con and HS groups. Arrows indicate antral follicles. (**B**,**C**) TUNEL staining and quantification of apoptotic cells in ovarian tissues. Apoptosis rate = TUNEL-positive cells/DAPI-stained cells. (**D**) Relative mRNA expression levels of apoptosis-related genes (*Bax*, *Caspase-3*, and *Bcl-2*) in ovarian tissues from the Con and HS groups. * *p* < 0.05; ** *p* < 0.01.

As shown in Table 3, the average ovarian weight of Hu sheep in the heat-stressed (HS) group was higher than that in the control group (1.40 ± 0.16 vs. 1.01 ± 0.13, *p* < 0.05). Further statistical analysis of follicle numbers in Hu sheep ovaries using hematoxylin and eosin (H&E) staining revealed that the numbers of primordial, primary, secondary, and mature follicles in the HS group were lower than those in the control group (*p* < 0.05). Meanwhile, compared to the control group, the number of antral follicles in the HS group was significantly increased (*p* < 0.05, Figure 4A). TUNEL assay results showed that the level of granulosa cell apoptosis in the ovarian tissues of the HS group was higher than that of the control group (*p* < 0.05, Figure 4B,C). In addition, the mRNA expression levels of pro-apoptotic genes (*Bax* and *Caspase-3*) in the ovaries of the HS group were higher, while the expression level of the anti-apoptotic gene (*Bcl-2*) was lower, compared to the control group (*p* < 0.05, Figure 4D).

### 3.4. Identification and Characterization of circRNAs in Ovarian Tissues of Hu Sheep Under HS

CircRNA-Seq analysis was performed on ovarian tissues from three randomly selected Hu sheep in each of the Con and HS groups (Table 4). cDNA libraries from six samples were sequenced using the Illumina HiSeq platform. Raw sequencing data were processed for quality control, filtering, and alignment. A total of 422,209,300 reads were obtained, of which 396,667,822 were successfully mapped to the reference genome. The GC content ranged from 47% to 52%.

**Figure 5:**
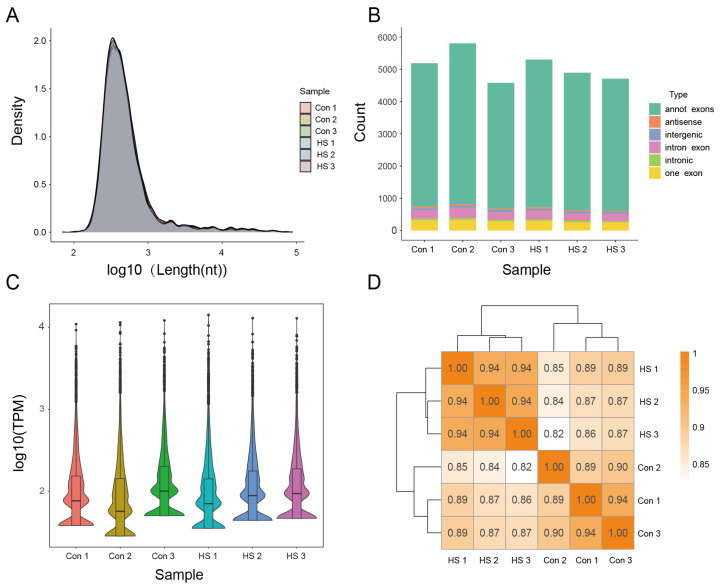
Quality assessment of circRNA-seq data. (**A**) Length distribution of circRNAs across samples. (**B**) Distribution of circRNA types across samples. (**C**) TPM density distribution of circRNAs in each sample. (**D**) Correlation analysis among samples.

Based on specific selection criteria, circRNAs were annotated, and their length distribution was analyzed. A relatively high proportion (21.22%) of circRNAs ranged from 300 to 400 nt in length (Figure 5A). The majority of circRNAs identified in Hu sheep ovarian samples (average = 87.55%) were exonic, primarily annotated as “annot_exons” (Figure 5B). Transcript abundance was quantified using transcripts per million (TPM), and TPM analysis indicated consistent circRNA expression levels and data density across samples (Figure 5C). High correlation was observed among ovarian tissue samples from both the control and heat-stressed groups, with correlation coefficients ranging from 0.82 to 1 (Figure 5D).

### 3.5. Differential Expression Patterns of circRNAs in Hu Sheep Ovaries Under HS

**Figure 6:**
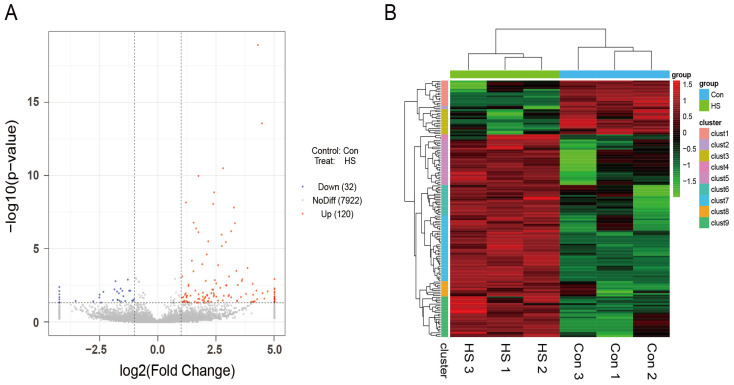
Screening and identification of differentially expressed circRNAs in ovarian tissue of Hu sheep. (**A**) Volcano plot of differentially expressed circRNAs. The x-axis represents log_2_FoldChange, and the y-axis represents −log_10_(*p*-value). The two vertical dashed lines indicate a two-fold change threshold, and the horizontal dashed line represents a *p*-value threshold of 0.05. Red dots indicate upregulated circRNAs, blue dots represent downregulated ones, and gray dots correspond to non-significantly changed circRNAs. (**B**) Hierarchical clustering of differentially expressed circRNAs. The x-axis represents circRNAs, with each column corresponding to a sample. Red indicates high expression, and green indicates low expression.

Differential expression analysis of circRNAs was conducted using edgeR 3.36.0, with thresholds set at |log_2_FoldChange| > 1 and *p* < 0.05. A total of 152 differentially expressed (DE) circRNAs were identified between the Con and HS groups, including 120 upregulated and 32 downregulated circRNAs (Figure 6A). Hierarchical clustering analysis revealed distinct expression patterns of DE circRNAs, clearly separating the Con and HS groups (Figure 6B).

### 3.6. Functional Enrichment Analysis of Source Genes of Differentially Expressed circRNAs in Hu Sheep Ovaries Under HS

**Figure 7:**
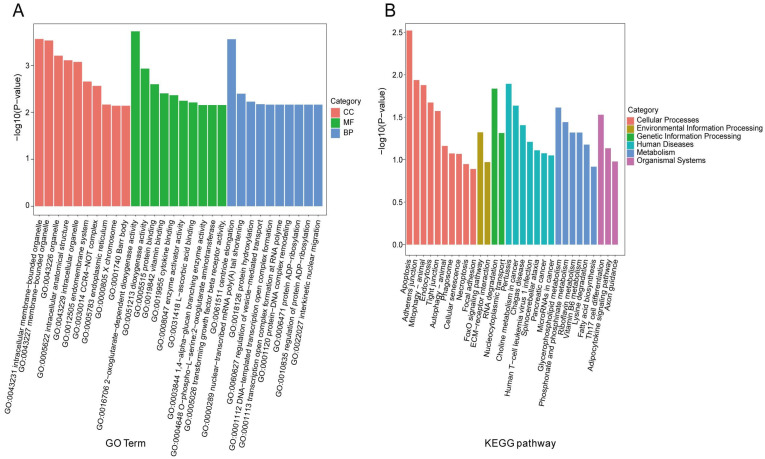
GO and KEGG enrichment analyses of source genes of differentially expressed circRNAs. (**A**) GO annotation of source genes of DE circRNAs. The x-axis represents GO level 10 terms, and the y-axis indicates the enrichment level as −log_10_(*p*-value) for each term. (**B**) KEGG enrichment analysis of source genes of DE circRNAs. The x-axis shows the pathway names, and the y-axis represents the enrichment level as −log_10_(*p*-value) for each pathway.

To explore the potential biological functions of DE circRNAs between the Con and HS groups, Gene Ontology (GO) and Kyoto Encyclopedia of Genes and Genomes (KEGG) enrichment analyses were performed on the source genes of 152 DE circRNAs. GO classification divided these genes into three categories: cellular component (CC), biological process (BP), and molecular function (MF) (Figure 7A). Most source genes were significantly enriched (*p* < 0.01) in terms such as intracellular membrane-bounded organelle (GO:0043231), membrane-bounded organelle (GO:0043227), organelle (GO:0043226), 2-oxoglutarate-dependent dioxygenase activity (GO:0016706), protein binding (GO:0005515), enzyme activator activity (GO:0008047), and centriole elongation (GO:0061511). KEGG pathway analysis (Figure 7B) revealed significant enrichment in Apoptosis (PATH:oas04210), Adherens junction (PATH:oas04520), Mitophagy—animal (PATH:oas04137), Tight junction (PATH:oas04530), FoxO signaling pathway (PATH:oas04068), Th17 cell differentiation (PATH:oas04659), and RNA degradation (PATH:oas03018).

### 3.7. RT-qPCR Validation of circRNAs

**Figure 8:**
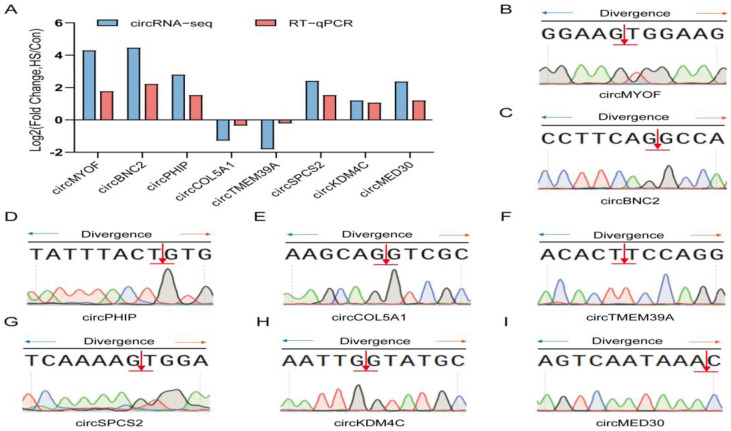
Validation of DE circRNAs in Hu sheep ovarian tissues. (**A**) Validation of circRNA-seq results. Eight DE circRNAs were randomly selected, and their expression levels were validated by qRT-PCR. (**B**–**I**) Sanger sequencing confirmed the back-spliced junction sequences of the specified circRNAs. The blue and orange arrows indicate divergent primers for amplifying the back-splice junction, and the red arrow marks the circularization site. Colored peaks below show the Sanger sequencing chromatogram (A: green, T: red, G: black, C: blue).

To validate the circRNA-seq results, eight differentially expressed circRNAs were randomly selected for analysis. RT-qPCR results confirmed that the expression patterns of these circRNAs were consistent with the sequencing data (Figure 8A). Subsequently, Sanger sequencing was performed to confirm the back-spliced junctions and verify the expected RNA sequences of the selected DEcircRNAs (Figure 8B–I). These findings further confirmed the reliability of the circRNA-seq results.

## 4. Discussion

With the continued intensification of global climate warming, heat stress (HS) is expected to cause long-term damage to livestock production worldwide [32]. As an important component of animal husbandry, sheep farming is particularly susceptible, with HS exerting adverse effects on sheep health [33]. Based on previous studies in other species showing that HS impairs reproductive function and that circRNAs play a regulatory role in this process, we hypothesized that circRNAs are involved in regulating the ovarian response of Hu sheep to HS. Therefore, in this study, we established a Hu sheep HS model and conducted circRNA sequencing of ovarian tissues to investigate how HS affects the physiological status of Hu sheep. Our results confirmed the hypothesis, demonstrating that HS induces ovarian dysfunction accompanied by altered circRNA expression, suggesting that circRNAs may play a potential regulatory role in the ovarian response to HS. These findings provide new insights into the molecular mechanisms underlying HS-induced impairments in the health and fertility of Hu sheep.

We first assessed the physiological response of Hu sheep to HS, which revealed significant stress indicators, confirming the successful establishment of the HS model. Hu sheep, as homeothermic animals, regulate their body temperature in response to environmental changes. When ambient temperatures exceed their thermoneutral zone, they experience heat stress (HS) [34]. The temperature–humidity index (THI) is a widely used indicator of HS, with values above 72 indicating stress [35]. Physiological indicators such as the respiratory rate (RR), pulse rate (PR), and rectal temperature (RT) also rise significantly under HS in a THI-dependent manner [36]. In this study, the average THI in the HS group was 91.54 ± 0.93. The RR, PR, and RT were significantly higher in the HS group than in the control group. Heat shock proteins (HSPs) help maintain cellular homeostasis under HS, and their expression reflects the animal’s stress response. Among them, *HSP70* is a key marker of thermotolerance [37,38]. Previous studies have shown that *HSP70* expression increases significantly in sheep during summer [39]. Consistent with this, serum HSP70 levels were significantly elevated in Hu sheep exposed to HS. Moreover, ovarian expression of *HSP60*, *HSP70*, and *HSP110* was markedly upregulated, while *HSP90* showed no significant change. These results confirm that the HS group experienced severe heat stress, which adversely affected their physiological state.

This study found that heat stress disrupts ovarian tissue morphology and increases granulosa cell apoptosis. In addition to promoting cell apoptosis, heat stress also significantly impairs redox balance and weakens the antioxidant defense capacity of the ovary. Previous studies reported reduced ovarian weight and increased granulosa cell apoptosis in sheep, mice, and cattle under HS [40,41,42,43]. Consistently, HS in Hu sheep significantly decreased ovarian weight, increased follicular granulosa cell apoptosis, upregulated pro-apoptotic genes (*Bax* and *Caspase-3*), and downregulated anti-apoptotic *Bcl-2*. Follicle numbers at all stages declined, while atretic follicles increased. In addition, previous studies have shown that heat stress (HS) negatively affects oxidative stress balance, leading to weakened antioxidant defenses in sheep and rabbits [44,45]. Consistent with these findings, our study demonstrated that HS reduced the levels of key antioxidant enzymes in Hu sheep, including catalase (CAT), glutathione peroxidase (GSH-Px), and total antioxidant capacity (T-AOC) (*p* < 0.05). These changes may result from excessive production of reactive oxygen species (ROS) in tissues induced by heat stress, which overwhelms the antioxidant defense system. Studies have shown that elevated ROS levels can directly damage cellular structures, increasing the demand for antioxidant enzymes [46]. However, HS may interfere with the expression and activity of antioxidant-related genes such as *CAT* and *GSH-Px*, which are essential for ROS clearance [47,48]. In our study, the downregulation of antioxidant genes (such as *SOD2*, *GPx*, and *CAT*) suggests that HS may suppress the transcriptional activation of these genes, reducing their enzymatic activity and consequently impairing overall antioxidant capacity. Given their regulatory potential, we next investigated circRNA expression profiles in the ovaries of Hu sheep under HS conditions. CircRNAs are a class of stable endogenous non-coding RNAs generated by the back-splicing of precursor mRNAs [49]. Increasing evidence suggests that circRNAs participate in various biological processes under physiological and pathological conditions in mammals through mechanisms such as acting as microRNA sponges, interacting with proteins, or encoding small peptides, thereby playing important roles in gene regulatory networks [50,51]. The ovary is a key reproductive organ in female mammals, and gene expression within the ovary directly influences reproductive capacity. Studies have shown that heat stress (HS) impairs ovarian function in livestock by disrupting critical signaling pathways, ultimately leading to reduced fertility [52]. However, the role of circRNAs in the ovarian response to HS remains largely unexplored. In this study, we performed circRNA-seq analysis on ovarian tissues from Hu sheep under HS and non-HS conditions to investigate the potential molecular mechanisms underlying the ovarian response to heat stress.

We identified and validated a total of 152 differentially expressed circRNAs. Functional enrichment analysis of the host genes of these circRNAs suggested that they may be involved in heat stress-induced apoptosis and stress responses through multiple signaling pathways. In our study, a total of 152 differentially expressed (DE) circRNAs were identified in ovarian tissues of Hu sheep between the control (Con) and heat stress (HS) groups, with 120 upregulated and 32 downregulated under HS conditions. Eight DE circRNAs were randomly selected for RT-qPCR validation, and their expression patterns were consistent with the circRNA-seq results, confirming the reliability of the sequencing data. GO and KEGG enrichment analyses of the host genes of DE circRNAs revealed significant enrichment in pathways related to apoptosis, mitophagy, and the FoxO signaling pathway, suggesting that circRNAs may regulate ovarian cell fate through these pathways. HS-induced germ cell apoptosis is a major factor contributing to reduced reproductive performance in livestock [53]. Abnormally high summer temperatures have been shown to trigger apoptosis in mammary and ovarian cells of dairy cows, impairing lactation and fertility [54,55]. Both mitophagy and the FoxO signaling pathway have been implicated as key regulators of heat stress-induced apoptosis [56,57], consistent with our enrichment findings. Additionally, GO analysis showed that the host genes of DE circRNAs were significantly enriched in functions such as 2-oxoglutarate-dependent dioxygenase activity, protein binding, dioxygenase activity, intracellular membrane-bounded organelle, and centriole elongation, which are closely associated with cellular homeostasis and responses to environmental stress.

Among the DE circRNAs, circBNC2 and circPHIP emerged as promising candidates, possibly contributing to ovarian cell stress responses and granulosa cell function under HS. Moreover, our data showed that both circBNC2 and circPHIP were significantly upregulated in the HS group. CircBNC2 has been reported to inhibit ovarian cancer cell proliferation and migration by targeting the miR-223-3p/FBXW7 axis [58]. *BNC2* is considered a key tumor suppressor gene in the ovary and plays a crucial role in regulating oxidative stress responses within ovarian cells [59]. *PHIP* is a potential candidate gene associated with egg production in chickens; its knockout significantly reduces progesterone (PROG) levels and inhibits granulosa cell proliferation in the ovary [60]. These findings suggest that circBNC2 and circPHIP may serve as important regulators in the ovarian response to heat stress.

## 5. Conclusions

In summary, our results demonstrate that heat stress (HS) negatively affects the physiological health and antioxidant capacity of Hu sheep, impairing follicular development and promoting the apoptosis of ovarian granulosa cells. Under HS conditions, a total of 152 differentially expressed circRNAs were identified, including 120 upregulated and 32 downregulated circRNAs. Functional enrichment analysis revealed that these circRNAs are involved in apoptosis, mitophagy, and the FoxO signaling pathway. These findings provide new insights into how circRNAs regulate ovarian function under heat stress and suggest that circRNAs such as circBNC2 and circPHIP may serve as potential biomarkers for heat stress resilience. However, further studies with larger sample sizes and broader age ranges are needed to validate these results and explore the potential mechanisms by which circRNAs modulate heat stress responses in livestock, which may contribute to the development of new strategies for enhancing livestock adaptability to climate change.

## Figures and Tables

**Table 1:** Dietary formula composition and nutritional level.

Ingredients (%)	Content	Nutritional Level	Content
Soybean meal	5	Dry Matter (DM)/%	58.52
Silage corn	48	Digestible Energy (DE)/(MJ/Kg)	15.18
Peanut vines	11	Crude Protein (CP)/%	8.88
Rice bran	8.3	Crude Fat (EE)/%	1.54
Dried cassava distillers’ grains	7.7	Crude Fiber (CF)/%	8.98
Premix ^1^	20	Crude Ash (Ash)/%	6.43
Total	100	Calcium (Ca)/%	0.28
		Total Phosphorus (TP)/%	0.19

Note: All values are expressed on an air-dry (as-fed) basis. ^1^ The Premix contains the following: Vitamin A 700,000 IU/kg, Vitamin E 900,000 IU/kg, Pantothenic acid 80 mg/kg, Biotin 4.0 mg/kg, Niacin 80 mg/kg, Iron (Fe) 500 mg/kg, Copper (Cu) 100 mg/kg, Manganese (Mn) 600 mg/kg, Zinc (Zn) 500 mg/kg, Iodine (I) 10 mg/kg, Selenium (Se) 2.25 mg/kg, Cobalt (Co) 2.25 mg/kg, Calcium (Ca) 12%, and Phosphorus (P) 2%.

**Table 2:** THI in sheep barns and physiological parameters of Hu sheep during the experimental period.

Items	Con Group (Mean ± SEM)	HS Group (Mean ± SEM)
THI	69.36 ± 1.9 ^b^	91.54 ± 0.93 ^a^
RR (times/min)	29.61 ± 1.03 ^b^	100.17 ± 1.75 ^a^
PR (beats/min)	72.04 ± 0.69 ^b^	132.83 ± 1.54 ^a^
RT (°C)	39.19 ± 0.04 ^b^	39.90 ± 0.05 ^a^

Note: THI, temperature–humidity index; RR, respiratory rate; PR, pulse rate; RT, rectal temperature; ^a,b^ different letters in the same row indicate significant differences between the two groups (*p* < 0.05).

**Table 3:** Number of follicles at different developmental stages in the ovaries of Hu sheep from the Con and HS groups.

Items	Con Group (*n* = 3)	HS Group (*n* = 3)
Primordial follicle	334.67 ± 40.55 ^a^	234.83 ± 50.65 ^b^
Primary follicle	123.33 ± 43.82 ^a^	68.50 ± 25.56 ^b^
Secondary follicle	47.17 ± 18.00 ^a^	19.17 ± 4.67 ^b^
Mature follicle	6.17 ± 1.47 ^a^	2.83 ± 0.75 ^b^
Antral follicle	82.50 ± 10.97 ^b^	101.50 ± 12.85 ^a^
Ovarian weight (g)	1.40 ± 0.16 ^a^	1.01 ± 0.13 ^b^

Note: ^a,b^ different letters in the same row indicate significant differences between the two groups (*p* < 0.05).

**Table 4:** Basic statistics of circRNA sequencing data.

Items	Total Reads	GC Content (%)	Total Mapped	Multiple Mapped	Unique Mapped
Con 1	77,664,144	52.45	72,839,894 (93.79%)	16,106,111 (22.11%)	56,733,783 (77.89%)
Con 2	71,290,888	50.56	67,100,196 (94.12%)	11,664,988 (17.38%)	55,435,208 (82.62%)
Con 3	65,846,436	50.91	62,029,594 (94.20%)	10,195,125 (16.44%)	51,834,469 (83.56%)
HS 1	66,346,110	50.68	62,322,041 (93.93%)	11,382,140 (18.26%)	50,939,901 (81.74%)
HS 2	65,255,110	48.61	61,110,183 (93.65%)	11,746,421 (19.22%)	49,363,762 (80.78%)
HS 3	75,806,612	47.53	71,265,914 (94.01%)	12,548,269 (17.61%)	58,717,645 (82.39%)

Note: Con represents ovarian tissues from the control group of Hu sheep, while HS refers to those from the heat-stressed group.

## Data Availability

The original contributions presented in the study are included in the article/Supplementary Materials. Further inquiries can be directed to the corresponding author.

## Supplementary Materials

The following supporting information can be downloaded at https://www.mdpi.com/article/10.3390/ani15142063/s1, Table S1: Gene-specific primer pairs used for RT-qPCR.
