# Supplementary material for: Impact of Heat Stress on Ovarian Function and circRNA Expression in Hu Sheep
Source: Animals (Basel). 2025 Jul 12;15(14):2063. doi: 10.3390/ani15142063 (PMC12291845; doi:10.3390/ani15142063)
Supplement: Supplementary file 1 [file animals-15-02063-s001.zip › animals-3716230-supplementary.pdf]

Supplementary Table S1. Gene-specific primer pairs used for RT-qPCR.

Genes	Sense primer (5'→3')	Antisense primer (5'→3')	Product length (bp)
HSP60	CCTTAATGCTACACGAGCTG	AACACCTGCATTCTTAGCAA	184
HSP70	CCCACGAAGCAGACGCAGAT	GCAGGTTGTTGTCCCGAGTCAT	106
HSP90	CAGTTCATTGGCTATCCCAT	TCTTCATCTGAGCCAACGTCT	164
HSP110	ACAAGCTGGAAGAACTAACGAA	AGTCAGCTGCTATCTTGGCAT	129
CAT	CCAATGTGCTTTCATCAGT	ATGCGGGAGCCATACTCAG	138
GPX1	GCAACCAGTTTGGGCATCAG	GCCATTCACCTCGCACTTTT	147
SOD2	TCACAGCATCTTCTGGACAA	TGCTCCTTATTGAAGCCAAG	116
BAX	ATGGGCTGGACATTGGACTT	ACTGTCTGCCATGTGGGTGT	143
BCL-2	CGAGTGGCGGCTGAAAT	GGTCTGCCATGTGGGTGTC	124
CASPASE3	TCAGGGAAACCTTCACGAGC	CCTCGGCAGGCCTGAATAAT	162
GAPDH	TCTCAAGGGCATTCTAGGCTAC	GCCGAATTCATTGTCGTACCAG	107
circMYOF	ACAACGTACCTGCACCTCTC	TCTTATCAGCGATGCCTCCAA	109
circBNC2	CTGGACCGTCTCTTCAGCGT	GTGCAGTTCACCAGTGTGCAG	131
circPHIP	TTCTTGGACACCTGTCGTCTG	AGCAAGTGATCGGGTGCTAA	103
circSPCS2	CGTCCAGTCTCAAAAGTGGAAGA	TTCACAGCTGATCCATCCCAT	72
circKDM4C	TTCCCATATGGCTACCATGC	GGTATTGCATACCAATTTAGCAACT	117
circMED30	TGGATCCCATTCCAGTAGAGC	ACACCGTTTGGCAGTTTATTGA	136
